# Ingestion of a single serving of saury alters postprandial levels of plasma n-3 polyunsaturated fatty acids and long-chain monounsaturated fatty acids in healthy human adults

**DOI:** 10.1186/1476-511X-11-95

**Published:** 2012-07-30

**Authors:** Zhi-Hong Yang, Hiroko Miyahara, Jiro Takeo, Masashi Katayama

**Affiliations:** 1 Central Research Laboratory, Tokyo Innovation Center, Nippon Suisan Kaisha, Ltd., 32-3 Nanakuni 1 Chome Hachioji, Tokyo, 192-0991, Japan

**Keywords:** Saury, n-3 PUFA, MUFA, Eicosapentaenoic acid (EPA), Docosahexaenoic acid (DHA)

## Abstract

**Background:**

Saury oil contains considerable amounts of n-3 polyunsaturated fatty acids (PUFA) and monounsaturated fatty acids (MUFA) with long aliphatic tails (>18C atoms). Ingestion of saury oil reduces the risk of developing metabolic syndrome concomitant with increases in n-3 PUFA and long-chain MUFA in plasma and organs of mice. We therefore evaluated changes in postprandial plasma fatty acid levels and plasma parameters in healthy human subjects after ingestion of a single meal of saury.

**Findings:**

Five healthy human adults ingested 150 g of grilled saury. Blood was collected before the meal and at 2, 6, and 24 hr after the meal, and plasma was prepared. Plasma levels of eicosapentaenoic acid, docosahexaenoic acid, and long-chain MUFA (C20:1 and C22:1 isomers combined) increased significantly throughout the postprandial period compared with the pre-meal baseline. Postprandial plasma insulin concentration increased notably, and plasma levels of glucose and free fatty acids decreased significantly and subsequently returned to the pre-meal levels.

**Conclusions:**

Our study suggests that a single saury meal may alter the postprandial plasma levels of n-3 PUFA and long-chain MUFA in healthy human subjects.

## Background

Increasing evidence from animal and human experiments has demonstrated that two particular long-chain n-3 polyunsaturated fatty acids (PUFA), i.e., eicosapentaenoic acid (EPA) and docosahexaenoic acid (DHA), have been associated with multiple positive health effects including improvement of obesity and diabetes mellitus [1], cardiovascular and neurodegenerative diseases [2,3], asthma [4], and inflammatory diseases [5]. Furthermore, we previously reported that a fish oil–derived concentrate containing long-chain monounsaturated fatty acids (MUFA), i.e., C20:1 and C22:1 isomers combined, alleviated metabolic syndrome partly by regulating genes involved in lipid metabolism, energy expenditure, and inflammation in obese mice [6]. In addition to n-3 PUFA, saury oil and pollock oil contain considerable amounts of long-chain MUFA [7,8]. Ingestion of saury oil or pollock oil countered the risk of developing metabolic syndrome and increases the plasma levels of n-3 PUFA and long-chain MUFA as well as the n-3/n-6 PUFA ratio in obese mice, suggesting a correlation between plasma levels of n-3 PUFA and long-chain MUFA and metabolic parameters [9,10]. However, limited information is available on the effect of saury meal ingestion on the plasma n-3 PUFA and long-chain MUFA accumulation in normal human subjects.

In this study, we investigated changes in plasma levels of EPA, DHA, and long-chain MUFA after ingestion of a single meal of saury. To our knowledge, this is the first report evaluating the effect of a single ingestion of saury on postprandial fatty acid composition and in plasma of healthy human subjects.

## Methods

Five healthy Asian adult volunteers (four Japanese and one Chinese; four males and one female; age range 30–40 years) in the experiment had normal serological measures, and they did not take any medications known to affect carbohydrate or lipoprotein metabolism or insulin secretion or its activity. All the participants lived in Japan, and regularly consumed traditional rice-based diets. Basal data of subjects are shown in Table 1. The study was approved by the ethics committee of Nippon Suisan Kaisha (Tokyo, Japan). Commercially prepared grilled saury (Maruko Foods, Shizuoka, Japan) was used in the study. After total lipid in the saury (150 g) was extracted by the Folch method [11], the fatty acid composition (Table 2) was determined as the methyl esters of fatty acids by gas-liquid chromatography. Measurement of the composition of the grilled saury was performed by Japan Food Research Laboratories (Tokyo, Japan), and the composition is shown in Table 3.

**Table 1:** Basal data of subjects before the experiment

Age (years)	35.1 ± 2.5
Height (cm)	169.2 ± 1.7
Body weight (kg)	60.8 ± 1.1
BMI (kg/m^2^)	21.2 ± 0.7
Plasma glucose (mg/dL)	96.8 ± 3.8
Plasma insulin (μIU/L)	1.9 ± 0.4
Plasma lipids profile	
TC (mg/dL)	191.2 ± 5.8
HDL-C (mg/dL)	67.1 ± 6.8
LDL-C (mg/dL)	99.4 ± 10.2
TG (mg/dL)	99.6 ± 14.3
FFA (mEq/L)	0.30 ± 0.05

**Table 2:** Major fatty acid composition of saury used in the study

**FA (%)**	
C14:0	6.22
C16:0	10.37
C18:0	1.80
C20:0	0.17
Total saturated FA	18.56
C16:1 n-7	1.94
C18:1 n-9	4.07
C20:1 n-9	12.55
C20:1 n-7	4.15
C22:1 n-11	20.08
C22:1 n-9	1.22
Total MUFA	44.01
C18:2n-6	1.32
C18:3n-6	0.16
C20:2n-6	0.26
C20:4n-6	0.42
Total n-6 PUFA	2.16
C18:3n-3	1.27
C20:3n-3	0.18
C20:5n-3	5.24
C22:5n-3	1.09
C22:6n-3	12.07
Total n-3 PUFA	19.85
n-3/n-6 PUFA ratio	9.19

FA: Fatty acids, MUFA: monounsaturated fatty acids, PUFA: polyunsaturated fatty acids.

**Table 3:** Composition of saury used in the study

**Component (g/100 g)**	
Moisture	48.9
Protein	25.9
Lipids	21.7
Ash	4.2
Carbohydrates	0.1
Energy (kcal/100 g)	299

The study was conducted after the subjects adhered to a 12-hr overnight fast, and fasting blood samples were collected at approximately 09:00 in the morning. Then, each subject ingested 150 g of grilled saury along with one rice ball within 15 min. Lunch was a medium-size plain noodle eaten between 12:00 and 12:30 at noon. Supper (medium-size rice or noodles) was ingested before 21:00 (19:00 ~ 20:00), and the subjects’ diet was restricted such that foods enriched in n-3 PUFA and/or MUFA were not consumed. No other meals or snacks between the three meals were ingested until the end of the experiment. Blood samples were collected at 2 hr and 6 hr after ingestion of the saury meal. Then, a final blood sample was collected at 24 hr after ingestion, and this was preceded by another 12-hr fast. Plasma was obtained by centrifuging each blood sample at 3000 rpm for 10 min at 4°C, and stored at −80°C until analysis. For each time point of the study, lipids were extracted from plasma using methanol/hexane, and the fatty acid composition was determined as described [9]. Postprandial plasma total cholesterol, high-density lipoprotein (HDL)-cholesterol, low-density lipoprotein (LDL)-cholesterol, triglycerides, free fatty acids, and glucose were determined enzymatically using commercially available reagent kits (Wako Pure Chemical Industries, Ltd., Osaka, Japan), and plasma insulin concentration was measured with an enzyme-linked immunosorbent assay kit (Morinaga Institute of Biological Science, Inc., Yokohama, Japan). The data are presented as mean ± SE, and the data between the fasting (time zero) and postprandial time points were compared. All statistical tests were performed using the Student's t-test, and statistical significance was considered as *P* < 0.05.

## Results and discussion

**Figure 1:**
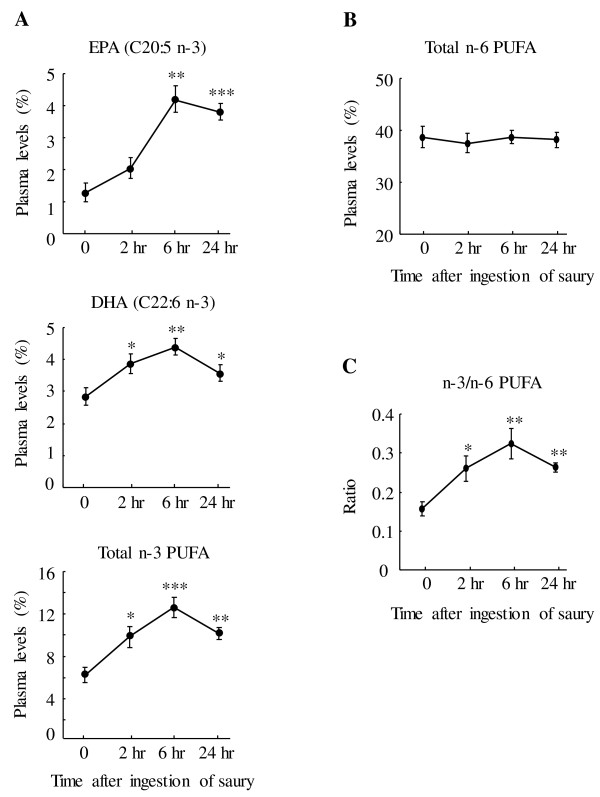
**Changes in levels of plasma PUFA.** Plasma levels (% of total plasma fatty acids) of EPA, DHA, and total n-3 PUFA (**A**), total n-6 PUFA (**B**), and n-3/n-6 PUFA ratio (**C**) are shown. The data were collected before and after the ingestion of a single saury meal. Values represent the mean ± SE, n = 5. *P < 0.05; **P < 0.01; ***P < 0.001 as compared to pre-ingestion values (time 0).

**Figure 2:**
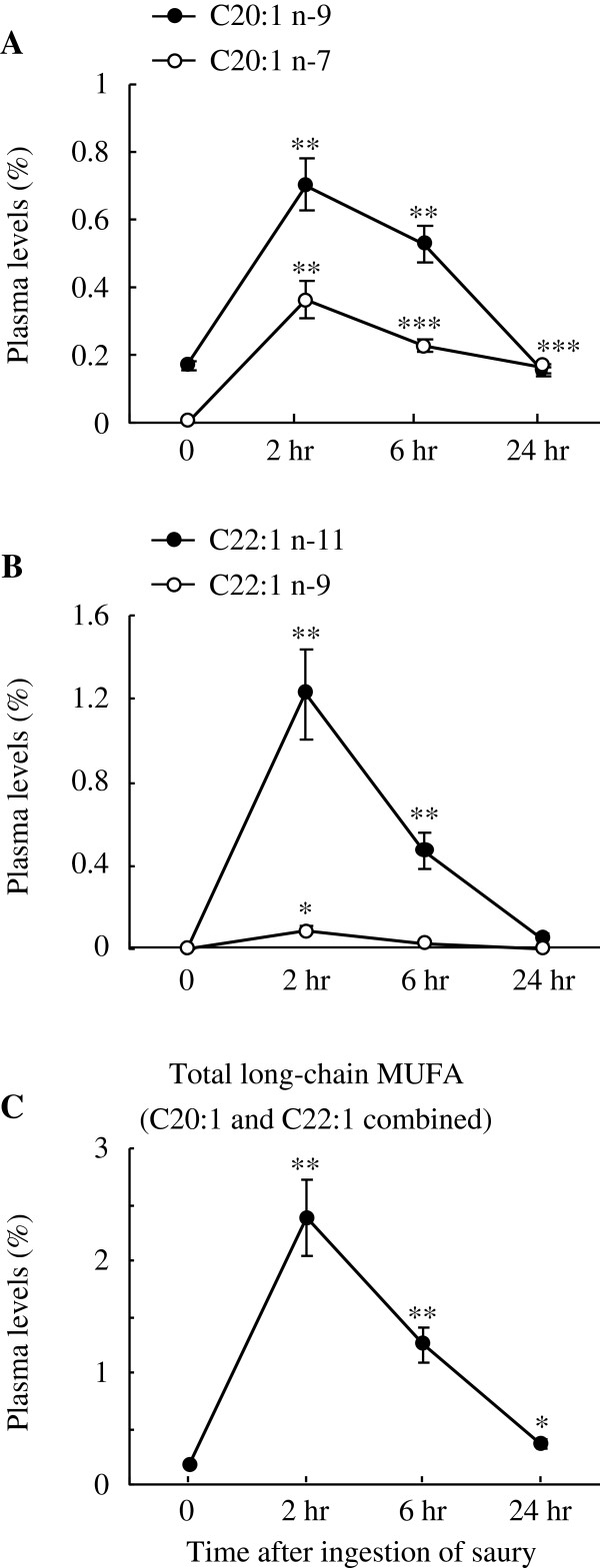
**Changes in plasma levels of MUFA with aliphatic tails >18C atoms.** Plasma levels (% of total plasma fatty acids) of C20:1 (n-9 and n-7) (**A**), C22:1 C20:1 n-9 (**B**), and total long-chain MUFA (C20:1 and C22:1 isomers combined) (**C**) are shown. The data were collected before and after the ingestion of a single saury meal. Values represent the mean ± SE, n = 5. *P < 0.05; **P < 0.01; ***P < 0.001 as compared to pre-ingestion values (time 0).

**Figure 3:**
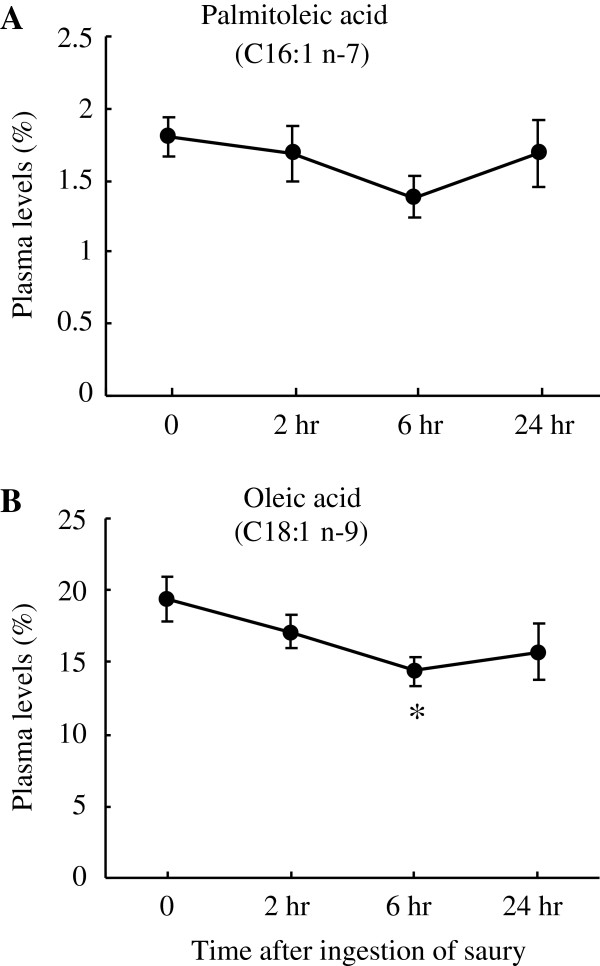
**Changes in plasma levels of MUFA with aliphatic tails <20C atoms.** Plasma levels (% of total plasma fatty acids) of palmitoleic acid (C16:1 n-7) (**A**) and oleic acid (C18:1 n-9) (**B**) are shown. The data were collected before and after the ingestion of a single saury meal. Values represent the mean ± SE, n = 5. *P < 0.05 as compared to pre-ingestion values (time 0).

The level of n-3 PUFA (EPA, DHA, and total n-3 PUFA) in plasma peaked at 6 hr after saury ingestion and then gradually declined (Figure 1a). At 24 hr post-ingestion, plasma EPA rose by 196% (*P* < 0.001), DHA rose by 25% (*P* < 0.05), and total n-3 PUFA rose by 65% (*P* < 0.01) compared with the pre-ingestion values. In contrast, plasma total n-6 PUFA levels remained essentially unchanged throughout the postprandial period (Figure 1b). Thus, the measured increase in n-3 PUFA level coupled with the lack of change in n-6 PUFA resulted in the notable increase (*P* < 0.05) in n-3/n-6 PUFA ratio from 2 hr to 24 hr after the saury meal (Figure 1c). Concerning plasma MUFA levels, long-chain MUFA C20:1 (n-9 and n-7) and C22:1 (n-11 and n-9) peaked at 2 hr post-ingestion (Figure 2a and b). Accordingly, plasma total long-chain MUFA (C20:1 and C22:1 isomers combined) increased from the fasting level of 0.17 ± 0.02% to 2.38 ± 0.34% at 2 hr after the saury meal and then declined sharply, although the value was still 1.2-fold higher (*P* < 0.05) than the basal value by the end of the study (Figure 2c). On the other hand, as shown in Figure 3, plasma levels of the shorter-chain MUFA (aliphatic tails <20C atoms) palmitoleic acid (C16:1 n-7) and oleic acid (C18:1 n-9) tended to decline throughout the experiment; the oleic acid level decreased by 26% (*P* < 0.05) at 6 hr after the saury meal. The long-chain MUFA gondoic acid (C20:1 n-9) and erucic acid (C22:1 n-9) are biosynthesized from oleic acid, and C20:1 n-7 is derived from palmitoleic acid by a series of chain-elongation reactions towards the carboxyl terminus [12]. Thus, the observed decrease in postprandial plasma levels of shorter-chain MUFA was possibly related to the elevated levels of circulating long-chain MUFA. Studies on the relationship between plasma fatty acid composition in Greenland Eskimos and the composition of Eskimo food have indicated that plasma levels of EPA/DHA as well as long-chain MUFA were found to reflect dietary intakes of these fatty acids [13,14]. The saury meal (150 g) used in the current study contained approximately 6 g of EPA and DHA combined and 12 g of C20:1 and C22:1 combined, which provided an abundant source of long-chain n-3 PUFA and long-chain MUFA. Furthermore, Osterud *et al.*[15] reported that the consumption of whale oil rich in n-3 PUFA (EPA and DHA) as well as long-chain MUFA (C20:1 and C22:1 isomers) by healthy subjects for 10 weeks significantly increased the serum levels of these fatty acids. It is therefore suggested that the sharp increases in plasma levels of long-chain n-3 PUFA and long-chain MUFA during the postprandial term in the current study were possibly due to the saury meal, which may possibly lead to the accumulation of these fatty acids in plasma after long-term ingestion of fish oil enriched in long-chain n-3 PUFA and long-chain MUFA. In a further study, we will investigate fatty acid alterations in response to dietary saury intake in a larger number of human subjects.

**Figure 4:**
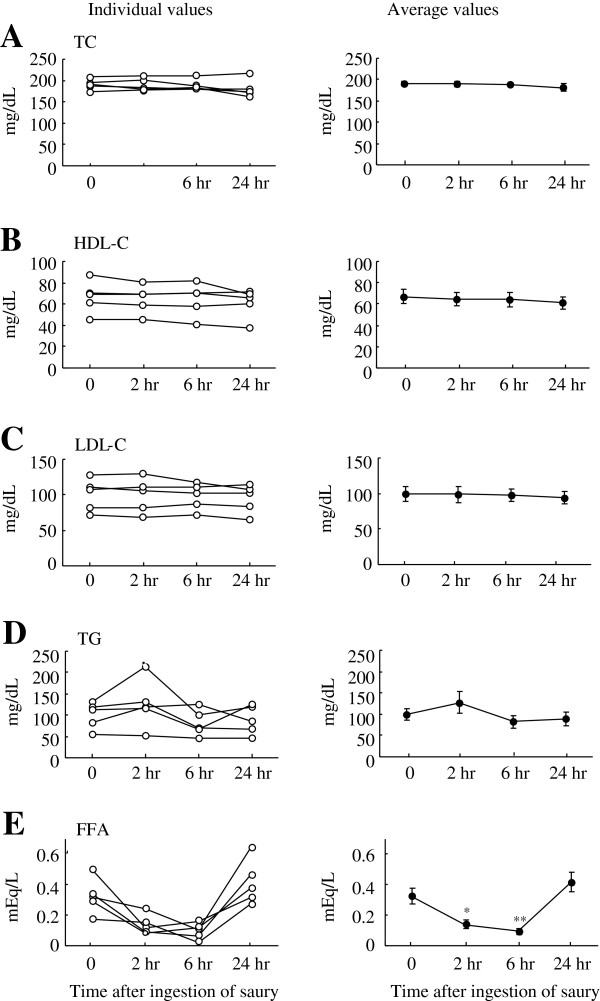
**Changes in postprandial plasma parameters related to lipid metabolism.** Individual (left panel) and average (right panel) levels of plasma TC (**A**), HDL-C (**B**), LDL-C (**C**), TG (**D**) and FFA (**E**) are shown. TC: total cholesterol, HDL-C: high-density lipoprotein cholesterol, LDL-C: low-density lipoprotein cholesterol, TG: triglyceride, FFA: free fatty acid. The data were collected before and after the ingestion of a single saury meal. Values represent the mean ± SE, n = 5. *P < 0.05; **P < 0.01 as compared to pre-ingestion values (time 0).

**Figure 5:**
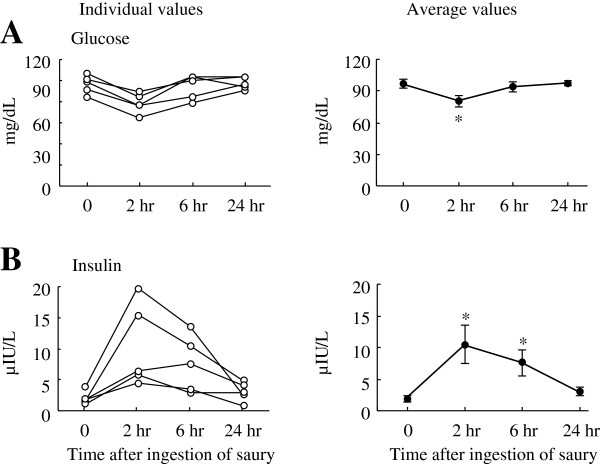
**Changes in postprandial plasma parameters related to glucose metabolism.** Individual (left panel) and average (right panel) levels of plasma glucose (**A**), and insulin (**B**) are shown. The data were collected before and after the ingestion of a single saury meal. Values represent the mean ± SE, n = 5. *P < 0.05 as compared to pre-ingestion values (time 0).

Plasma concentrations of free fatty acids decreased from a baseline value of 0.32 ± 0.05 mEq/mL to 0.14 ± 0.03 mEq/mL (*P* < 0.05) at 2 hr and 0.09 ± 0.02 mEq/mL (*P* < 0.01) at 6 hr after the saury meal and then returned to the basal level by 24 hr (Figure 4e), although there were no differences in plasma levels of total cholesterol (Figure 4a), HDL-cholesterol (Figure 4b), LDL-cholesterol (Figure 4c) and triglyceride (Figure 4d) throughout the experimental period. Plasma glucose (97 ± 4 mg/dL, basal) decreased by 17% (*P* < 0.05) at 2 hr post-ingestion and then returned to basal levels (Figure 5a). In contrast, plasma insulin concentration increased from 1.9 ± 0.4 μIU/L to 10.5 ± 3.1 μIU/L (*P* < 0.05) by 2 hr and to 7.6 ± 2.0 μIU/L (*P* < 0.05) by 6 hr in response to the saury meal and returned to the basal level by the end of the study (Figure 5b). It has been well documented that insulin plays a crucial physiological role in postprandial glucose homeostasis and in inhibiting adipose tissue lipolysis, which may lead to reduced release of fatty acids into the blood stream [16,17]. Thus, the hyperinsulinemia after the saury meal may have caused the observed acute decreases in plasma glucose and free fatty acids. It has been reported that dietary fats of varying degree of unsaturation exert different effects on postprandial glucose/lipid homeostasis, and a MUFA-rich diet improves insulin sensitivity acutely [18]. Nevertheless, we could not exclude the possibility that other components in the meal were involved in the observed changes in the postprandial plasma parameters. To clarify the effectiveness of saury on mitigating the postprandial insulin response, it is necessary to directly compare the saury oil and other dietary fats.

## Abbreviations

DHA: docosahexaenoic acid; EPA: eicosapentaenoic acid; MUFA: monounsaturated fatty acids; PUFA: polyunsaturated fatty acids.

## Competing interests

The authors declare that they have no competing interests.

## Authors' contributions

Conceived and designed the experiments: ZHY, HM, JT and MK. Performed the experiments: ZHY and HM. Analyzed the data and wrote the paper: ZHY.
