# Correction: ingestion of a single serving of saury alters postprandial levels of plasma n-3 polyunsaturated fatty acids and long-chain monounsaturated fatty acids in healthy human adults

**DOI:** 10.1186/1476-511X-11-151

**Published:** 2012-11-08

**Authors:** Zhi-Hong Yang, Hiroko Miyahara, Jiro Takeo, Masashi Katayama

**Affiliations:** 1 Central Research Laboratory, Tokyo Innovation Center, Nippon Suisan Kaisha, Ltd., 32-3 Nanakuni 1 Chome Hachioji, Tokyo 192-0991, Japan

##  

After publication of this manuscript [1], we noted that one of the monounsaturated fatty acid isomers had been mislabelled in the results, and in Figure 2A and Table 2.

In the Results and discussion, the sentence, “Concerning plasma MUFA levels, long-chain MUFA C20:1 (n-9 and n-7) and C22:1 (n-11 and n-9) peaked at 2 hr post-ingestion (Figure 2a and b)” should read, “Concerning plasma MUFA levels, long-chain MUFA C20:1 (n-11, n-9 and n-7) and C22:1 (n-11 and n-9) peaked at 2 hr post-ingestion (Figure 2a and b)”.

The figure and table have also been corrected (Figure 1, Table 1).

**Figure 1:**
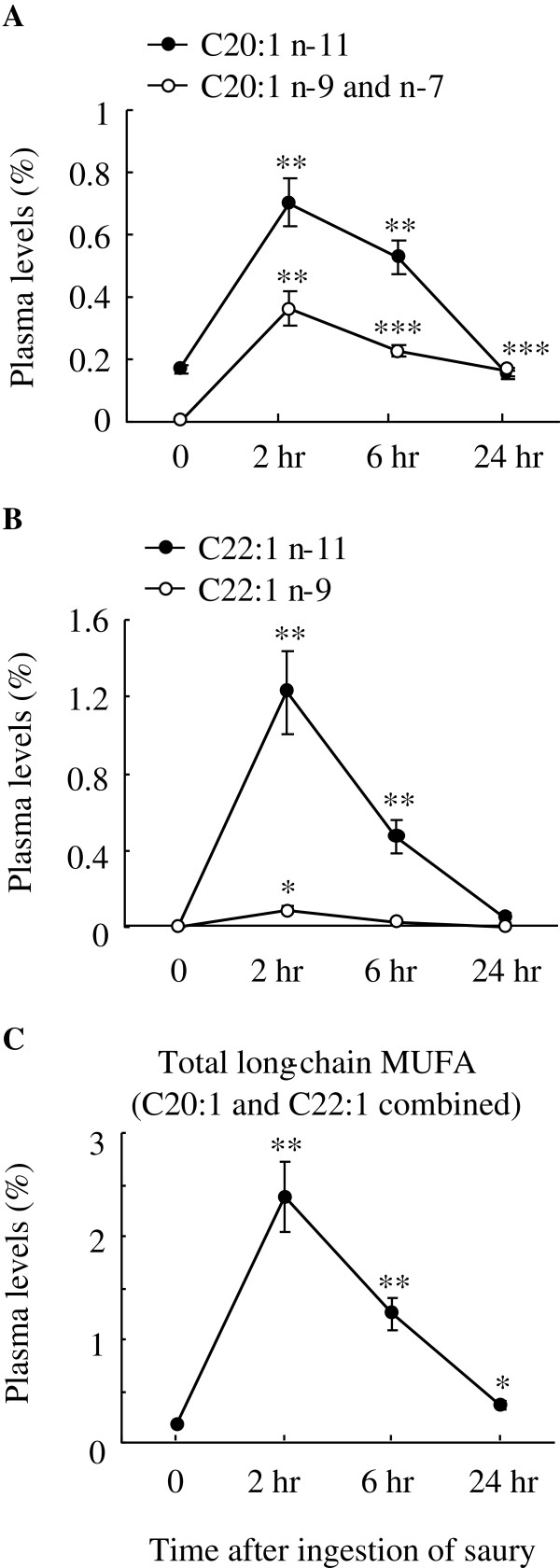
**Changes in plasma levels of MUFA with aliphatic tails >18C atoms.** Plasma levels (% of total plasma fatty acids) of C20:1 (n-11, n-9 and n-7) (**A**), C22:1 C20:1 n-9 (**B**), and total long-chain MUFA (C20:1 and C22:1 isomers combined) (**C**) are shown. The data were collected before and after the ingestion of a single saury meal. Values represent the mean ± SE, n = 5. *P < 0.05; **P < 0.01; ***P < 0.001 as compared to pre-ingestion values (time 0).

**Table 1:** Major fatty acid composition of saury used in the study, correction shown in red

**FA (%)**	
C14:0	6.22
C16:0	10.37
C18:0	1.80
C20:0	0.17
Total saturated FA	18.56
C16:1 n-7	1.94
C18:1 n-9	4.07
C20:1 n-11	12.55
C20:1 n-9	4.15
C22:1 n-11	20.08
C22:1 n-9	1.22
Total MUFA	44.01
C18:2n-6	1.32
C18:3n-6	0.16
C20:2n-6	0.26
C20:4n-6	0.42
Total n-6 PUFA	2.16
C18:3n-3	1.27
C20:3n-3	0.18
C20:5n-3	5.24
C22:5n-3	1.09
C22:6n-3	12.07
Total n-3 PUFA	19.85
n-3/n-6 PUFA ratio	9.19

FA: Fatty acids, MUFA: monounsaturated fatty acids, PUFA: polyunsaturated fatty acids.

We apologise for this error, which was due to a misreading of the gas chromatography reading.
